# HTLV-1, ATLL, severe hypercalcaemia and HIV-1 co-infection: an overview

**DOI:** 10.11604/pamj.2018.30.61.13238

**Published:** 2018-05-28

**Authors:** Abdullah Ebrahim Laher, Osman Ebrahim

**Affiliations:** 1 Department of Emergency Medicine and Department of Critical Care, Faculty of Health Sciences, University of the Witwatersrand, 5 Jubilee Road, Parktown, Johannesburg, 2193, South Africa; 2 Department of Internal Medicine and HIV clinic, Life Brenthurst Hospital, Johannesburg, South Africa

**Keywords:** HIV-1, HTLV-1, ATLL, refractory hypercalcaemia, severe hypercalcaemia

## Abstract

HIV and HTLV (Human T-ymphotropic Virus) are the only known retroviruses responsible for causing infection in humans. HTLV-1 and HIV-1 are frequent co-pathogens, however, despite its potential for accelerated progression of HIV disease and the risk of developing adult T-cell lymphoma/leukemia (ATLL), HTLV-1 is seldom considered for investigation in the HIV-1 positive individual. Severe/refractory hypercalcaemia, unresponsive to conventional calcium lowering therapy may complicate up to 70% of cases of ATLL. In addition, HTLV-1 and ATLL have both been associated with a rise in dysfunctional CD4 lymphocytes, thereby conveying a false sense of immune competence in the HIV-1 infected individual.

## Introduction

Human T-lymphotropic virus 1 (HTLV-1) was first described in the 1980's, but in actual fact is an "ancient" pathogen that has infected humans for many centuries [1]. Besides human immunodeficiency virus (HIV), HTLV is the only other retroviruses known to cause disease in humans [2]. HTLV-1 and HIV-1 are frequent co-pathogens [3]. With the current HIV epidemic affecting predominantly sub-Saharan Africa [4], there is renewed interest in this organism. Many clinicians treating patients with HIV-1 are unaware of the possibility of HTLV-1 co-infection and its associated potential for accelerated progression to AIDS as well as the risk of developing adult T-cell lymphoma/leukemia (ATLL) or HTLV-associated myelopathy / tropical spastic paraparesis (HAM/TSP) [5]. In the HIV positive patient presenting with severe/refractory hypercalcaeima, the likelihood of occult ATLL and HTLV-1 co-infection must be considered. The triad presentation of HTLV-1, ATLL and severe/refractory hypercalcaemia has been described in case reports [6-9]. However, the true prevalence of this presentation is unknown, largely due to misdiagnosis and underreporting as HTLV-1 is seldom considered for investigation in HIV infected individuals [10]. Current areas of uncertainty surrounding HTLV-1 co-infection include its impact on the progression of HIV disease and the initiation of antiretroviral therapy in countries where access to these drugs are restricted [3,11]. We (authors) have recently managed and been made aware of a number of HIV-1 infected individuals that had presented with refractory hypercalcaemia secondary to HTLV-1 associated ATLL. In this article, we review aspects of this presentation with the aim of improving awareness of the concurrent presence of HTLV-1 co-infection in the HIV-1 infected individual, especially in sub-Saharan Africa and other high HIV prevalence settings. We also aim to emphasize the potential for accelerated progression of HIV-1 disease, the difficulties in interpreting the CD4 cell count when deciding on the initiation of (antiretroviral therapy) ART or when monitoring HIV-1 disease progression as well as the pathogenesis and basic management of refractory hypercalcaemia in the HIV-1 infected individual with concurrent HTLV-1/ATLL.

## Methods

**Figure 1:**
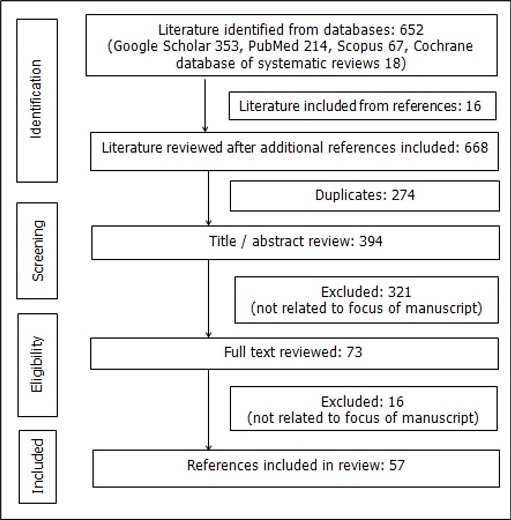
Flow diagram for literature search

A comprehensive literature search strategy was conducted using the following electronic databases: cochrane database of systematic reviews, Google Scholar, PubMed, Scopus and Web of Science (June 2017). The following search terms were used; HIV, HTLV, human T-cell lymphotropic virus, ATLL, Adult T-cell leukemia/lymphoma, hypercalcaemia.' The citations of literature generated by the search were also reviewed for any additional literature. The search was restricted to all literature relating to the topic that was published in English and after the year 1970. Articles included in the review met the following criteria: i) publications were scientific in nature and included journal articles, conference abstracts as well as book chapters ii) the full text of the publication was available. The search yielded 652 articles as follows: google scholar 353, pubmed 214, scopus 65 and cochrane database of systematic reviews 18. The publications were screened for both duplicates and content. Sixteen other articles were identified from the citations provided by the selected publications. Two hundred and seventy four duplicate entries and another 321 entries were removed as they were not directly related to the topics identified for discussion. The remaining 73 publications had full text available. Fifty seven references were included in the final draft. Details of the above are summarized in Figure 1.

## Current status of knowledge

**HTLV-1**: HTLV-1 was first described in the USA in 1980 [12] and independently again in 1982 in Japan [13,14]. In endemic regions such as Japan, the Carribean, South America and sub-Saharan Africa, the reported prevalence of HTLV-1 amongst the general population is between 0.5% and 20% [10,15]. Like HIV, HTLV-1 is a retrovirus that not only also infects CD4 T-lymphocytes but is similar in structure and route of transmission to the HIV virus [16]. HTLV-1, however, is not as easily transmitted as HIV-1 [17]. HIV-1 and HTLV-1 have also been shown to co-infect the same CD4+ cell [18]. Ninety five percent of individuals infected with HTLV-1 remain asymptomatic. The other 5% manifest with ATLL or HTLV associated myelopathy/tropical spastic paraparesis (HAM/TSP) [19,20]. In the Caribbean HTLV-1 is commonly associated with refractory and persistent staphylococcal and streptococcal cutaneous infections [21]. There have also been case reports linking HTLV-1 to the presentation with uveitis, arthritis, sjogrens syndrome and strongyloides stercoralis hyperinfection [22]. In terms of diagnosis, antibodies to HTLV-1 can be detected with a screening ELISA test. A positive result must be confirmed with detection of gene products on Western blot analysis. PCR has a poor sensitivity for the detection of viral RNA as a detectable plasma viral load is seldom present in most cases [5]. Antiretroviral agents such as lamivudine, zidovudine and raltegravir have not shown clear benefit in patients with HTLV-1 infection [23,24].

**ATLL**: HTLV-1 has oncogenic potential and is invariable present in almost all cases of ATLL, a neoplasm of mature CD4 T-lymphocytes [25]. Not surprisingly, the prevalence is highest in HTLV-1 endemic regions described above [26]. The median age of diagnosis is in the 6^th^ decade of life in HIV uninfected individuals, but has been shown to be much earlier in HIV co-infected individuals [17]. Several forms of ATLL have been described [27]. More than half of patients present with the acute form which is characterised by organomegally, lymphadenopathy and a leukaemic picture on laboratory analysis. About half of these patients also present with skin lesions and hypercalcaemia which has a tendency to be severe/refractory. In the chronic form, patients present with chronic lymphocytosis that develops over months to years, skin lesions, small volume lymphadenopathy and no hypercalcaemia. The smouldering form is the most benign. Patients are usually asymptomatic or present with skin rashes that is generally responsive to the application of a topical corticosteroid ointment. In the lymphoma form, patients manifest with organomegally, raised LDH, occasional hypercalcaemia but no blood involvement [27,28]. In terms of therapy, the best results are achieved with a combination of zidovudine, alpha interferon and chemotherapy. However the overall prognosis of ATLL is poor [29]. The median survival of the acute and lymphoma forms is less than 1 year, whilst the prognosis with the other subtypes is slightly better [30]. The chemotherapeutic regimen of choice comprising VCAP-AMP-VCEP (VCAP-vincristine, cyclophosphamide, doxorubicin, prednisone; AMP-doxorubicin, ranimustine, prednisone; VECP-vindesine, etoposide, carboplatin, prednisone) has been associated with a trend towards an improved 3 year survival. However, due to significant toxicity, not many patients are able to complete therapy with this regimen and besides, ranimustine and vindesine are not widely available in many countries including South Africa and the USA [31]. The addition mogamulizumab, a humanised anti-CC chemokine receptor 4 antibody has also shown to improve outcomes, but again is not widely available [32].

**HTLV-1 and HIV-1 co-infection**: Sub-Saharan Africa, with more than two-thirds of global HIV cases, boasts the highest prevalence of HIV in the world. South Africa, having more people with HIV than any other country in the world, has an estimated 18.9% HIV prevalence amongst individuals 15 years of age and older [4]. The co-prevalence of HTLV-1 and HIV-1 has been reported as 4-23% in asymptomatic HIV infected persons and 5-28% in individuals with AIDS [10,33-35]. In a study conducted in 1993 in Northern KwaZulu-Natal (KZN) that comprised 1018 subjects, the HTLV-1 seroprevalence was reported as 2.6%, whilst the HIV-1 seroprevalence was 3.5%. The relative risk of co-infection with HIV-1 and HTLV-1 was noted as 1.16 (95% CI 1.08-1.24) [36]. Another South African study that included asymptomatic urban and rural black subjects from the Free State region, reported a HTLV-1 seroprevalence of 1.6%. Six percent of HIV seropositive patients in this study were also co-infected with HTLV-1 [37]. More recently and of concern, 24% of HIV-positive sera tested positive for the presence of HTLV-1/2 IgG antibodies in a study that included 170 HIV-positive plasma specimens from the Limpopo province of South Africa [33]. HTLV-1 may also induce HIV-1 viral replication. Whilst it is uncertain whether HTLV-1 is associated with a shortened survival [38,39], studies have suggested an accelerated progression of HIV disease and worsened outcomes after infection with opportunistic organisms in co-infected individuals [11,40]. Persons co-infected with both HIV and HTLV-1 have a 2.5 fold higher risk of contracting tuberculosis, especially in countries with a high prevalence of tuberculosis, such as sub-Saharan Africa and Brazil. These patients also have a higher associated morbidity and mortality risk [41,42].

**Interpretation of the CD4 cell count in individuals with HTLV-1/ATLL and HIV-1 co-infection**: The CD4 cell count must be interpreted with caution in the HIV-1 positive individual with concurrent ATLL and or HTLV-1 co-infection. Both HTLV-1 and ATLL have been associated with a rise in CD4 cells. Whilst reactive polyclonal proliferation of infected CD4 cells accounts for the increase in HTLV-1 infection, clonal expansion of CD4 lymphocytes may additionally contribute to the relative rise in individuals with concurrent ATLL [1,27,43]. A Mozambican study demonstrated that HTLV-1 co-infected individuals were 7 times more likely to present with a CD4 cell counts > 500 cells/μl [44]. In another study that comprised 184 HIV infected individuals from Nigeria, the mean CD4 cell count was noted as 742 cells/μl in HTLV-1-positive subjects compared to 380 cells/μl in HTLV-1-negative subjects [10]. HIV and HTLV-1 both independently induce a functional modification of CD4 and other T cell populations [45,46] that is characterized by an increase in naïve cells and a higher level of cell activation when compared to uninfected persons [11]. Progress of immunosuppression in individuals with ATLL/HTLV-1 co-infection is evidenced by an increasing HIV-1 viral loads and clinical progression of AIDS despite a stable or rising CD4 cell count [47]. Therefore, in the HIV infected individual with ATLL and or HTLV-1 co-infection, the absolute CD4 cell count may mask the degree of immunosuppression. These individuals may actually present a more advanced stage of HIV disease and are at increased risk of acquiring opportunistic infections and presenting with other immune suppression related clinical manifestations despite similar CD4 cell counts as HIV positive individuals without ATLL/HTLV-1 co-infection [3,48,49]. Although South Africa has recently adopted the World Health Organization (WHO) recommendation with regards the initiation of ART in all individuals living with HIV, irrespective of the CD4 cell count or the degree of immune suppression [50,51], the falsely raised CD4 cell count in individuals with concurrent ATLL/HTLV-1 co-infection may have implications with regards to the initiation of antiretroviral therapy in other resource limited-settings such as sub-Saharan Africa which bears the brunt of the global HIV burden [3,4]. Hence CD4+ counts in HIV-1/HTLV-1 co-infected individuals is considered an unreliable marker of immunologic competence and the need to initiate ART. Similarly quantitative HTLV-1 proviral load (PVL) has also been reported to correlates poorly with disease severity and the need to initiate ART's [41].

**ATLL associated hypercalcaeima**: The pathogenesis of hypercalcaemia in patients with ATLL has been associated with elevated serum macrophage colony-stimulating factor (M-CSF) levels, over expression of receptor activator of nuclear factor kappa B ligand (RANKL), ATLL infiltration into the bone marrow and perhaps parathyroid hormone-related peptide (PTH-rP) secretion [52]. In contrast, to patients with non-Hodgkin's lymphoma where the incidence of hypercalcaemia on presentation is < 3% [53], severe and refractory hypercalcaemia has been shown to complicate ATLL in approximately 70% of cases and is one of the chief causes of early mortality [54]. Therefore the possibility of occult ATLL and HTLV-1 co-infection must be considered as part of the differential diagnosis in the HIV positive patient presenting with hypercalcaemia. Basic calcium lowering therapy such as the administration of high volume intravenous fluids, loop-diuretics, corticosteroids and intravenous bisphosphonate is frequently inadequate in sufficiently lowering calcium levels. Literature on the application of haemodialysis in the management of refractory hypercalcaemia is surprisingly scarce with no available consensus guidelines. Case reports and case series have reported success with haemodialysis using a low calcium concentrate dialysate (1-1.5 mmol/L) [55,56]. Recently denosumab, a fully humanised monoclonal antibody has been associated with success in 2 cases of bisphosphonate refractory hypercalcaemia [57].

## Conclusion

In conclusion, more studies are required to determine the prevalence of HTLV-1 and its impact on CD4 lymphocyte counts (both absolute and relative), HIV viral load and AIDS progression in co-infected individuals in sub-Saharan Africa and other regions with a high HIV burden. The clinician managing the HIV positive patient presenting with hypercalcaemia must consider the possibility of occult ATLL and HTLV-1 co-infection.

### What is known about this topic

- The co-prevalence of HTLV-1 and HIV-1 has been reported as 4-23% in asymptomatic HIV infected persons and 5-28% in individuals with AIDS;
- The CD4 cell count must be interpreted with caution in the HIV-1 positive individual with concurrent ATLL and or HTLV-1 co-infection as both HTLV-1 and ATLL have been associated with a rise in CD4 cells;
- Severe and refractory hypercalcaemia has been shown to complicate approximately 70% of cases of ATLL and is one of the chief causes of early mortality.

### What this study adds

- Many clinicians treating patients with HIV-1 are unaware of the possibility of HTLV-1 co-infection and its associated potential for accelerated progression to AIDS as well as the risk of developing adult T-cell lymphoma/leukemia (ATLL);
- The possibility of occult ATLL and HTLV-1 co-infection must be considered as part of the differential diagnosis in the HIV positive patient presenting with hypercalcaemia.

## Competing interests

The authors declare no competing interest.
